# Food-Derived Bioactive Peptides in Human Health: Challenges and Opportunities

**DOI:** 10.3390/nu10111738

**Published:** 2018-11-12

**Authors:** Subhadeep Chakrabarti, Snigdha Guha, Kaustav Majumder

**Affiliations:** 1 Bureau of Nutritional Sciences, Food Directorate, Health Products and Food Branch, Health Canada, Ottawa, ON K1A 0K9, Canada; subhadee@ualberta.ca; 2 Department of Food Science and Technology, University of Nebraska-Lincoln, Lincoln, NE 68588-6205, USA; sguha3@unl.edu

**Keywords:** bioactive peptides, enzymatic hydrolysis, fermentation, peptide absorption, oral bioavailability, functional foods

## Abstract

Recent scientific evidence suggests that food proteins not only serve as nutrients, but can also modulate the body’s physiological functions. These physiological functions are primarily regulated by some peptides that are encrypted in the native protein sequences. These bioactive peptides can exert health beneficial properties and thus are considered as a lead compound for the development of nutraceuticals or functional foods. In the past few decades, a wide range of food-derived bioactive peptide sequences have been identified, with multiple health beneficial activities. However, the commercial application of these bioactive peptides has been delayed because of the absence of appropriate and scalable production methods, proper exploration of the mechanisms of action, high gastro-intestinal digestibility, variable absorption rate, and the lack of well-designed clinical trials to provide the substantial evidence for potential health claims. This review article discusses the current techniques, challenges of the current bioactive peptide production techniques, the oral use and gastrointestinal bioavailability of these food-derived bioactive peptides, and the overall regulatory environment.

## 1. Introduction

The physicochemical roles of proteins in foods, apart from serving as dietary nutrients, are being increasingly acknowledged. Many of these physicochemical roles of naturally occurring dietary proteins are carried out by peptide sequences encrypted inside the parent protein, which exert their actions when released, either enzymatically, during food processing, or by microbial fermentation [1,2]. Bioactive peptides are defined as peptide sequences within a protein that exert a beneficial effect on body functions and/or positively impact human health, beyond its known nutritional value [3]. These peptides can regulate important bodily functions through their myriad activities, including antihypertensive, antimicrobial, antithrombotic, immunomodulatory, opioid, antioxidant, and mineral binding functions [4,5,6,7].

Different activities of the bioactive peptides are governed by the sequence of the amino acids, as they would interact with other proteins in the body and modulate natural processes [8]. Although the structure and functional relationship of bioactive peptides are not well established, most of them share some common properties. For instance, most of these peptides contain 2 to 20 amino acids and are generally rich in hydrophobic amino acids [3,9]. Thus, over the last few years, there has been an increased scientific interest in finding distinct bioactive peptide sequences that can reduce or prevent the risk of chronic diseases and provide immune protection [2]. Thus, the use of bioactive peptides has gained much interest as nutraceuticals [10] and functional foods [11]. As a result, much research has been dedicated recently to the processing and generation of bioactive peptides from food products, and the previously under-utilized protein-rich by-products of the food industries [12,13]. The bioactivity of these peptides could be tested through in vitro bio-chemical assays, cell culture, in vivo studies via animal models, and human clinical trials. While the research related to the development of food-derived bioactive peptide-based nutraceuticals is gaining momentum, the ability to translate these new findings into practical or commercial use remains delayed. The major reasons behind this delay are (1) lack of scalable and consistent methods of producing bioactive peptides from different food or non-food sources; (2) general lack of understanding of gastrointestinal stability or absorption of these peptides; (3) lack of knowledge of their mechanisms of actions, and (4) lack of proper clinical trials to provide substantial evidence for potential health claims. Thus, the scope of this review includes the challenges pertaining to manufacturing/processing, oral use and/or bioavailability, and the regulatory environment governing use of bioactive peptides.

## 2. Production of Bioactive Peptides

A diverse array of plant and animal food proteins has been used for extracting bioactive peptides [7]. The most widely used animal proteins are from eggs, milk (casein and whey), and meat proteins. Bioactive peptides from plant sources are typically from soy, oat, pulses (chickpea, beans, peas, and lentils), canola, wheat, flaxseed, and hemp seed. Furthermore, proteins from marine sources have also been used, for instance, fish, squid, salmon, sea urchin, oyster, seahorse, and snow crab [3,9]. In the manufacturing process, food proteins from various sources are first digested with an enzyme, and then the biological activity of the whole hydrolysate is evaluated, followed by a series of activity-guided purification and identification, so as to find the most potent sequence. However, the activity-guided identification and purification of bioactive peptides is time-consuming, and often studies do not provide enough rational behind the selection of enzymes. To overcome these issues, a quantitative structure–activity relationship (QSAR) and bioinformatics based in-silico method is often used to predict the yield of bioactive peptides from food protein sources [14]. However, this method works best only when we have the complete sequence of a food protein and the structure and functional properties of the peptides are known. Unfortunately, there is lack of understanding of such structure–functional properties and hence, researchers are still using the traditional activity-guided methods to search for bioactive peptides from food proteins. The following section highlights the basic bioactive production techniques.

### 2.1. Production Methods

Bioactive peptides from food proteins can be produced either by enzymatic hydrolysis (using proteolytic enzymes from either plants or microbes), hydrolysis with digestive enzymes (simulated gastrointestinal digestion), or by fermentation using starter cultures. Some studies also used a combination of these methods to produce peptides with a biological activity [2]. Furthermore, bioactive peptides can also be synthesized chemically, as the amount of these peptides found in the nature is very low, and there is a constant increasing commercial interest of producing synthetic bioactive peptide [15]. However, it is doubtful if purely synthetic peptides would be considered as food or nutrients, and fit within the scope of this review. Therefore, in this review, we primarily discuss the enzymatic hydrolysis and fermentation, and briefly introduce the chemical synthesis process for producing bioactive peptides.

#### 2.1.1. Enzyme Hydrolysis

In the enzymatic hydrolysis method, the protein of interest is subjected to enzymatic treatment at a specific pH and temperature. The advantages of this method are that it is easy to scale up and generally has a shorter reaction time than microbial fermentation [1]. For instance, in a study by Gobbetti et al., angiotensin converting enzyme (ACE)-inhibitory peptides were generated from the fermentation of milk, using the strains *Lactobacillus lactis* ssp. *cremoris* and *Lactobacillus delbrueckii* ssp. *bulgaricus*, each for 72 h separately [16]. This was in contrast to the study by El-Fattah et al., where bioactive peptides with ACE-inhibitory activities were produced from the hydrolysis of milk using protease (*Aspergillus oryzae*) for only 1 h [17]. More than one protease can also be used one after the other to generate shorter peptides, however, the temperature and pH would need to be optimized for each of the proteases [1]. Furthermore, the choice of protease used and the time of enzymatic hydrolysis could be important in deciding the type of peptides generated. For example, rice proteins hydrolyzed with bacillolysin showed a stronger anti-inflammatory and anti-tyrosinase activities compared with those hydrolyzed by subtilisin [18]. On the other hand, samples hydrolyzed with leucyl and papain aminopeptidase and cysteine endopeptidase showed the least activity [18]. Similarly, different enzymes generated bioactive peptide-rich hydrolysates from bovine muscle and porcine plasma with divergent taste profiles, as observed in a recent study [19]. Several studies have also used in-vitro simulated gastrointestinal digestion technique to produce bioactive peptides from food proteins [20,21,22,23]. In such methods, the researchers have tried to identify the activity of the peptides that may be produced in our body after consuming a particular food or food-protein.

#### 2.1.2. Fermentation

Fermentation involves the culturing of microorganisms, such as yeasts, fungi, or bacteria, on the protein of interest in order to hydrolyze the protein into shorter peptides with their own enzymes. The bacteria usually needs to be in the exponential growth phase before they are harvested, washed, and added to glucose containing sterile distilled water, which ultimately serves as the starting inoculum for the protein substrate [24,25]. The degree of hydrolysis depends on the fermentation time, microbial strain, and the protein source. For instance, Ahn et al. showed that the ACE-inhibitory activity of whey protein derived peptides fermented with *Lactobacillus brevis* was stronger than those fermented with *L. casei*, *L. lactis*, *L. plantarum*, and *L. acidophilus* [26]. Similarly, Sanjukta et al. demonstrated that soybean proteins fermented by *Bacillus subtilis* MTCC5480 produced a higher degree of hydrolysis compared with *B. subtilis* MTCC1747 [27]. Co-cultures using different combinations of bacteria, yeasts, and fungi can also be used to modulate the hydrolysis processes [28].

### 2.2. Production Issues

The classical approach of producing bioactive peptides is to find a suitable protein source, followed by its hydrolysis, using either enzymes or by microbial fermentation, so as to generate peptides with a potential bioactivity. This would be followed by the identification of the peptide sequences and confirmation of the bioactivity. However, much of the published literature on bioactive peptides have not taken a systematic approach to optimize the multiple parameters affecting the production and purification of these peptides [29]. Hanke and Ottens suggested that “one factor at a time” and “trial and error” methods are obsolete and are being taken over by systemic design of experiments (DOE) approaches. DOE methods require the knowledge of the critical process parameters (CPP) that would affect the critical quality attributes (CQA) [30]. Certain CPPs, with respect to the production of bioactive peptides, requires knowledge of the starting material (protein content of a food and seasonal variabilities), the enzyme (optimal temperature and pH, purity, specific activity, and substrate specificity), and finally the process conditions (time, temperature, and enzyme to substrate ratio). On the other hand, certain CQAs may be recognized for the peptide fractions or the protein hydrolysates [29]. For example, Cheung and Li-Chan applied a Taguchi’s L_16_ (4^5^) fractional factorial design to study the effect four CPPs on three CQAs (degree of hydrolysis, ACE-inhibitory effect, and bitterness) of the protein hydrolysate obtained from the by-products of shrimp processing. The use of this DOE approach enabled the assessment of the protein hydrolysates using only 16 unique experiments, which were generated under conditions linked with the combination of the four CPPs. This was in contrast to the use of either a full factorial design with 256 unique experiments, or a one-factor-at a time experiment, where one factor is changed, keeping the other three constant. However, the use of Taguchi’s methods and other DOE approaches are more beneficial in other disciplinary sectors, while its recognition in the field of food-derived bioactive peptides has been limited [31].

Furthermore, Kopf-Bolanz et al. suggested that processing can affect the peptide profiles and cause protein degradation when present in food matrices such as dairy products [32]. During thermal processing, besides Maillard reactions, oxygen- and carbon-based radicals can be generated, which could lead to the oxidation of proteins, peptides, and carbohydrates [33]. A number of studies have been reported for food-derived bioactive peptides from the hydrolysis of protein isolates or protein concentrates in isolation, rather than a direct hydrolysis of the whole food [34,35]. However, it is critical to consider the food matrix, which may also influence the hydrolysis reaction. Foods contain many naturally occurring compounds, such as lipids, carbohydrates, and secondary metabolites (like quinones), which interacts with the proteins in the matrix, and thus can affect the type of peptides generated upon hydrolysis. Schiff base reactions between reducing sugars and peptides are well established. Peptides undergo reactions with reactive oxygen species, oxidized lipids, and aldehydes, as well as decarboxylation, deamination, and nitration reactions. All of these could potentially affect the availability of the peptides within the food matrix [33]. For instance, in a study by Lacroix and Li-Chan, the whey protein constituents were hydrolyzed individually by pepsin. Among them, α-lactalbumin hydrolysate showed the highest dipeptidyl peptidase IV (DPP-IV) inhibitory activity, although the specific peptides responsible for the inhibition were not identified. However, in a subsequent study, in order to identify the peptides responsible for the DPP-IV inhibition, it was found that the most potent anti-DPP-IV peptides were from β-lactoglobulin rather than α-lactalbumin. This suggests that co-existence of different proteins in a particular food matrix might induce conformational changes during commercial production, which might in turn affect the susceptibility and accessibility of the peptide bonds during digestion [29].

In-silico prediction methods, such as QSAR, use knowledge of the activity and structure of peptides present in the databases and literature. It can be used to predict the sequences of the peptides likely to have any bioactivities, their structural–functional relationships, specific location of the peptides within the parent protein, and the possible mechanism of action [36]. However, even though there is much data on food peptides and the enzymes required to release them from the source proteins, the majority describe the endogenous bioactive peptides, which are of physiological relevance, instead of those that are obtained from food [37]. Furthermore, the information that is available in the databases often involves well-characterized and purified proteolytic enzymes, in comparison with the commercially used enzymes for food processes, which are less substrate specific and of variable purity [38].

### 2.3. Commercialization Challenges and Quality Assurance

Once the peptides are produced, either through classical ways or by in-silico methods, the next step is to confirm the bioactivity of the peptides. However, unlike synthetic drug molecules, which are single entities, the target bioactive peptides isolated from foods are usually a mixture of peptides. The purification of these peptides to 99% purity would not only increase the cost to unacceptable levels and reduce the yields, but would also eliminate any beneficial additive or synergistic effects with other peptides present in the whole hydrolysate. Furthermore, bioactive peptides are generally hydrophobic, and thus they are less soluble at higher concentrations. Indeed, Li-Chan suggested preparing formulations of several different bioactive peptides, each having a low concentration, but conferring similar bioactivity levels, to address this problem [29].

Food proteins are often hydrolyzed using enzymes such as trypsin, pepsin, chymotrypsin, bromelain, ficain, or papain. Although there are several advantages to using enzymatic hydrolysis, such as the absence of residual toxic chemicals and organic solvents in the final product, the use of the enzymes on an industrial scale highly increases the cost of the production. One solution to that is to use cheaper enzyme sources such as by-products of the meat industry (i.e., pancreases of animal origin) [39]. Secondly, a mixture of peptides is generated during in vitro enzymatic hydrolysis, depending on the complexity of the starting material. This in turn makes the process of purification time-consuming and challenging; in some cases, each of the peptide may require a complex purification protocol [40].

On the other hand, naturally occurring peptides have many advantages compared with the peptides produced by enzymatic hydrolysis, as these peptides are perceived to be safe [40,41]. However, the lack of technology at a larger scale and very expensive purification techniques are some of the limitations for the commercialization of extracting naturally occurring bioactive peptides from food sources [40]. Thus, research should focus on addressing the above-mentioned challenges associated with production methods for commercial applications of these food-derived bioactive peptides.

## 3. Oral Use of Bioactive Peptides: Challenges and Considerations

As these peptides are derived from food, they are generally considered more “natural”; hence, perceptions of acceptance are likely to be higher. Yet, their use as orally ingested products also presents special challenges and consequences.

### 3.1. Taste

The oral intake of food and medicinal products is fundamentally dependent on taste. Taste is often the body’s first response to an orally ingested substance. We eat things that taste good and reject those with bitter or other unpleasant tastes. It is believed to be an evolutionary response, developed over millennia to avoid toxic or rancid substances [42]. As such, it is vital for orally taken products to have a favorable taste profile. Protein hydrolysates and individual peptides often fail on these grounds, as a significant number of these products are bitter, which may limit their acceptability [43,44]. A number of studies have identified factors such as increasing molecular weight, presence of hydrophobic amino acids at the C-terminal, presence of certain amino acid sequences, and degree of electrical charge with a propensity towards bitterness (reviewed in [45]). However, the molecular mechanisms of bitterness and its regulation are not completely understood; hence, the modification rather than prevention of the bitter taste may be a more feasible option in many instances.

Traditionally, bitterness modification (also called “debittering”) has been approached through methods to reduce levels of these bitter-tasting peptides. One of the procedures involves the further hydrolysis of the product (bioactive peptide or protein hydrolysate, generated by initial enzymatic hydrolysis) by enzymes, to reduce the content of any bitter-tasting peptides [46,47,48]. While reasonably effective, this process can be expensive because of the costs of additional enzymes, and it also risks inadvertently destroying the very bioactive properties that made the preparation valuable in the first place. The alternative option has been to “screen out” bitter peptides from a complex mixture involving one or more techniques, such as gel separation, alcohol extraction, chromatography on silica gel, and isoelectric precipitation (reviewed in [49]). While each of these methods has its benefits, the time and expense added to a commercial production scheme are often considerable. Besides, the lack of a comprehensive structure–activity relationship between taste and molecular structure further impedes on the successful application of such a method to a growing array of bioactive peptides derived from a range of food proteins.

An alternative approach is to modify, modulate, or mask the offending taste, instead of trying to screen it out using the addition of taste-modifying agents, such as various sugars, salts, and nucleotides, as suggested by Leksrisompong et al. [50]. Starter cultures of *Lactobacillus* added to the proteins during hydrolysis have been touted as another taste modifying agent that could be acceptable because of their widespread use in fermented food products since ancient times [11,49]. Deamidation, the removal of amino groups by specific enzymes, is another option that has been shown to increase umami-tasting peptides, which also contributes to the masking of an existing bitter taste [51]. Interestingly, a recent study demonstrated that specific peptides from beef protein hydrolysates could block the bitter taste receptor T2R4 and directly inhibit bitter taste perception instead of simply masking it [52]. This is an exciting discovery of bioactive peptide/s blocking bitter taste sensation (which could be derived from other bioactive peptides), and further exemplifies the versatility of these peptides in offering novel solutions to persistent problems.

### 3.2. Digestion

Orally ingested substances are metabolized by various digestive enzymes, starting in the oral cavity, continuing in the stomach, and finally in the small and large intestines. A number of proteolytic enzymes are present in the human body, and their actions can irreversibly alter the peptide profile of such products. Indeed, many bioactive peptide preparations were initially produced by mimicking the digestive environment in the gastrointestinal (GI) tract, with protease treatment yielding the “active” peptides out of the native protein structure (reviewed in [3,7,53]). Being generated through simulated digestion, some bioactive peptides, such as the egg protein derived tripeptide IRW, are naturally resistant to digestive enzymes [54]. This is a huge advantage in delivering bioactives through the oral route, as a lack of digestion in the GI tract ensures increased bioavailability and a better chance of exerting a significant effect on the body’s physiology. On the other hand, some peptides such as LKPNM, derived from enzymatic digestion of bonito fish protein, are further metabolized into their active components in the GI tract (LKP, an anti-hypertensive tripeptide is released from LKPNM), which then exert the intended biological action upon absorption into the systemic circulation. This could be considered analogous to a pro-drug, which undergoes metabolism to yield the active ingredient [55].

The skeptic may now question the need to generate peptides (or hydrolysates) through in vitro enzymatic procedure, as all orally ingested proteins are digested anyway in the GI tract. While a definitive yes/no answer is unlikely, it is plausible response is that an industrial scale digestive method may generate a different profile of bioactive peptides, which could then be characterized through chemical and biological assays to define their physiologic effects. The use of different enzymes can yield bioactive peptides from the same source protein with diverse biological functions, which could be tailored to different physiological (and potentially pathological) needs. This could be due to the different enzymes cleaving the same source protein at different sites, as well as the subsequent digestion of initially generated peptides, both of which contribute towards the generation of distinct peptide repertoires. Indeed, a study by Offengenden et al. used a range of commercially available enzymes, used singly or in combination, to generate a number of chicken collagen hydrolysates with different actions on proliferation, extracellular matrix deposition, and resistance to inflammation in osteoblastic cells [56]. Similar studies have been done on hydrolyzed proteins sourced from egg and milk proteins [57,58]. Another potential benefit is the unmasking of specific bioactive sequences, which may not be accessed/generated/released under normal digestive processes. For example, a study by Jahandideh et al. showed that enzymatically pre-digested fried egg preparations significantly reduced blood pressure in spontaneously hypertensive rats, while the lack of such pre-digestion completely abolished this antihypertensive effect [59].

Finally, protein hydrolysates containing an array of peptides may undergo GI tract digestion to yield a different set of peptides, the biological effects of which are still incompletely understood. Surprisingly, a study of casein (a milk protein) hydrolysates in infant formula has shown a reduced variety of casein peptides compared to formula with intact casein. However, the functional significance of these differences remain unclear [60]. Indeed, comparison studies of infant formula with intact and (extensively) hydrolyzed protein have shown similar effects on growth and tolerance, suggesting the possibility of a functional overlap and/or redundancy among different casein peptides [61].

### 3.3. Absorption

Absorption from the GI tract is essential for a bioactive peptide to exert any systemic biological actions downstream. Traditionally, it was believed that all peptides and proteins were digested down to their constituent amino acids, and only these amino acids were capable of absorption across the intestinal epithelial barrier. Indeed, the absorption of larger entities such as peptides and proteins were only considered as pathological phenomena, and a key culprit in food allergies! However, it is apparent now that many peptides do cross the intestinal epithelium under normal conditions, enter into the circulation, and exert systemic effects (reviewed in [61,62]).

Several mechanisms have been postulated to explain the intestinal uptake of peptides from the GI lumen, as detailed in the review by Lundquist et al. [63]. Briefly, the key mechanisms are as follows: paracellular transport through intercellular tight junctions; direct penetration of the epithelial cell membranes; endocytosis/phagocytosis by cells; and last, but not least, active transport by specific carrier proteins. Each of these mechanisms may occur alone or in association with others, while the same peptide may utilize one or more different approaches, adding to the complexity. A number of approaches have been tried in order to estimate and enhance the intestinal absorption of proteins and peptides, a brief overview of such potential solutions will be given here.

Paracellular transport is mediated through one or more tight junction proteins [64]. Two different approaches have been tested to increase peptide absorption by modulating the permeability of these junctions. The use of absorption enhancers, either covalently bound to the bioactive peptide or just used in conjunction, can enhance the uptake of the bioactive molecules [65,66]. However, this increased permeability is hard to modulate, and uncontrolled permeability changes could lead to localized inflammation and long-term damage to the intestinal epithelium [67]. An alternative method is targeting the myosin light chain phosphorylation process, which regulates cellular shape changes and intercellular junction integrity. Under physiological conditions, the myosin light chains are held in a state of equilibrium between its phosphorylated and dephosphorylated forms. The myosin light chain kinase phosphorylates its target, while the myosin light chain phosphatase exerts an opposite effect by dephosphorylating it. A higher level of phosphorylation would “open up” the intracellular tight junctions, allowing for greater access to peptides [68,69]. Thus, the transient inhibition of myosin light chain phosphatase, which shifts the balance towards increased phosphorylation, has been touted as an alternative approach to enhance peptide transport through tight junctions, but its clinical efficacy is yet to be verified [63].

The direct penetration of the cell membrane is a property of many peptides, and some bioactive peptides may utilize this mechanism to cross the intestinal epithelium on their own [70]. In addition, highly cell-permeable peptides, such as HIV-Tat and Penetratin, could be covalently conjugated to various bioactive peptides for a more efficient delivery [71,72]. However, further research may be needed to determine the nature of the membrane crossing abilities and the factors (peptide composition as well as external issues like pH and presence of minerals) that modulate such actions [73].

Endocytosis and/or transcytosis by epithelial cells could be enhanced if bioactive peptides are encapsulated within the carrier molecules known to be targets of such processes [74]. A number of approaches such as the use of liposomes or nanomaterials have been investigated for this purpose. Typically, such microencapsulation helps to protect the bioactive peptide inside, while addition of other molecules on the outer surface of the particles helps with its adhesion, localization, and eventual uptake by the intended target cells [75]. A number of different approaches have been utilized to enhance the intestinal uptake, including use of bacterial toxins, antibody fragments, and polysaccharides [76,77,78].

Finally, many peptides are selectively transported by specific transporters such as Pept1, an active transporter of oligopeptides. Studies undertaken by several research groups have demonstrated the key regulatory roles played by these transporter proteins on the transport of exogenous bioactive peptides [79,80,81]. Hence, the pharmacological modulation of these molecules may offer one of the more plausible avenues for regulating peptide absorption in the GI tract (reviewed in [82,83]). Future therapeutic approaches could involve modulation at the level of these transporters, allowing for further fine tuning of the intestinal uptake of the beneficial peptides [84].

In summary, the absorption of intact peptides, either alone or as part of a protein hydrolysate, is an exciting area of research that is critical for the successful oral use of these compounds. As an examination of the specific mechanisms and therapeutic approaches in greater detail is beyond the scope of this article, the interested reader is referred to two excellent reviews by Muheem et al. and Lundquist et al. [62,63].

### 3.4. Local Effects

Peptides do not necessarily need to be absorbed from the GI tract in order to exert biological effects. The GI tract is a large organ by itself, and local actions of bioactive peptides is an area of growing interest. Chronic diseases of the GI tract, especially inflammatory bowel diseases (IBD) in all their various manifestations, are a major cause of morbidity in the developed world. Current pharmacological treatments offer limited benefits at best, and require a lifelong adherence to therapeutic regimens, with their attendant cost and side-effects. As such, alternative therapies are an attractive idea to manage these diseases, and there exists the potential for locally-acting peptides (and protein hydrolysate preparations), given orally, to step into the void. A large number of food peptides have already been validated for their anti-inflammatory and anti-oxidant properties [5], which make them, either alone or in combination, theoretically well-suited for management of IBD cases [85,86]. While clinical data in humans is still lacking, a study in cats demonstrated the efficacy of commercially available hydrolyzed protein preparation in resolving pre-existing IBD [87]. Similarly, a recent study showed the beneficial effects of an egg shell membrane hydrolysate to attenuate the experimental GI tract inflammation in mice, further supporting the therapeutic potential of orally taken peptides acting in situ [88].

In conclusion, the oral use of bioactive peptides and protein hydrolysates offer a number of unique advantages and challenges that require further efforts in research and development targeted towards different aspects such as palatability, digestion, and sites of action. The development of these peptides for health promoting and therapeutic purposes would have to take into account these factors when devising strategies for oral usage.

## 4. Regulatory Environment for Bioactive Peptides

The regulatory environment includes the laws, regulations, and licensing systems that govern the manufacture, import, export, and sale of regulated products. In the context of biomedical and food industries, it involves various aspects of foods, drugs, and other products with effects on health and nutrition. Most advanced economies have robust regulatory regimens that ensure the safety and (where applicable) efficacy of such products, in order to protect the well-being of their citizens [89]. Given the novelty and potential health and nutritional roles of food derived bioactive peptides and protein hydrolysates, it is critical to understand and engage with the regulatory system/s in place in order to successfully translate the discoveries from the laboratory to the real world. While regulatory regimens do vary across different national and regional jurisdictions [90,91,92,93], certain common themes observed are discussed here.

### 4.1. Food or Drug?

The first issue is whether a product is a food or a drug. As bioactive peptides are obtained from food proteins and are purported to have health benefits, this could be more complicated than it seems at first glance. For example, if a peptide is derived from milk and reduces high blood pressure, is it a food, drug, or both? However, national regulatory systems are quire decisive about the food versus drug classification, and a product could be placed as either a food or a drug with little overlap between them.

The general principle is to focus on their intended use. If a product is taken as a food (i.e., the primary use is to gain sustenance and/or nutrition), it should be considered a food. On the other hand, if the primary use is to mitigate a disease or improve a bodily function, it is a drug. The latter category includes both natural health products and pharmaceuticals, as mentioned later. Indeed, the Canadian Food and Drugs Act (F&D Act) clearly defines “food” and “drug” based on their intended usage profile [94].

However, it can be argued that in some instances, the food/drug dichotomy is less clear cut and there are a number of product categories that straddle the divide, despite being legally defined as either “food” or “drug”. As a growing number and variety of natural products become available for general use, it may be reasonable to consider such products as part of a food-to-drug continuum, with traditional foods at one end and dietary supplements (or, natural health products) and/or pharmaceuticals on the other.

### 4.2. Traditional Foods

While the concept of bioactive peptides is relatively new; many such products have been in widespread use since time immemorial. Across different cultures and continents, people have used foodstuff like yoghurt/cheese/kefir (milk protein derived peptides), pickles (peptides from fermented fruit or vegetable proteins), and fermented soybean products (tempeh, tofu, and natto), which are rich sources of food peptides, many with well-known bioactive properties [95,96]. Being widely known for their culinary use and regarded as safe to eat, these products have the least regulatory requirements. As long as these are prepared in a sanitary environment and use food grade chemicals (e.g., enzymes and processing aids), no special action is needed.

### 4.3. Novel Foods

This category includes foods that lack a history of safe use or those that have undergone novel processing methods that significantly change their nutritional or safety aspects. The Canadian Food and Drug Regulations (FDR) describing novel foods also include genetically modified organisms under its aegis [97]. For bioactive peptides and hydrolysates, the first sub-category may involve an unusual (or less widely known/used) protein source, while the second sub-category may involve the use of “new” enzymes, bacteria (for fermentation), and any number of chemical/physical methods used to generate, protect, or preserve an array of peptides. One or more applications may be needed to seek approval for usage and the consequent marketing of foods containing such ingredients. In Canada, novel food applications may involve the final food product, or it may pertain to a processing aid or a bacterial strain. In the United States, both foods and food-making processes can be covered under “generally recognized as safe” (GRAS), a process used to allow for both novel products and procedures [98,99].

### 4.4. Functional Foods

While traditional and novel foods are widely sold, there are restrictions on health-related claims pertaining to these products. There is a growing tendency within both the scientific and industry communities to promote the concept of a “functional food”, that is, a food endowed with specific health/medical functions over and above its nutritional role [95,100,101]. Indeed, a recent search on PubMed for “functional food” merited over 4000 hits, compared to ~2500 for “bioactive peptides” and ~1100 for “protein hydrolysates” (personal observation, May 2018). Despite the wide usage of this term, its legal validity remains unclear. Countries like Canada and the United States do not provide any legal status for “functional food”, although Health Canada had defined such a product [102]. In contrast, Japan has long maintained a regulated category of “food for specific health uses” (FOSHU), which may be the closest approximation to functional foods as a legal concept [102,103,104]. Outside Japan, such usage has remained uncommon, and so this may not be a viable regulatory option for bioactive peptide-based products in most markets in the near future.

### 4.5. Food for Special Uses

There are a number of special regulatory requirements for foods for special uses, which may involve those for medical conditions (e.g., low-energy, high-energy, and low in particular amino acids) or those intended for vulnerable populations. The commonest example is infant formula (for those aged 0–12 months). Most jurisdictions use stringent regulatory standards to protect infants’ nutritional needs; as such, it is one of the common regulated foods sold all over the world. Globally, infant formula is regulated by Codex Alimentarius standards (e.g., Codex STAN72-1981), under the auspices of the World Health Organization (WHO) and the Food and Agriculture Organization (FAO) [105]. Nationally, many countries follow these international standards, while others, such as Canada and the United States, use their own standards for infant formula [97,106]. Given the putative role of hydrolysis in reducing the allergenicity of milk proteins, milk protein derived bioactive peptides are already a key component of many infant formulae sold across the world [107,108]. Indeed, this may well be one of the major instances of enzymatically pre-digested food proteins being used in a mass-marketed product. With the rising interest in both hypoallergenic and vegan diets, it is likely that peptide-rich plant protein hydrolysates (e.g., those from soy or rice proteins) would become an alternative source for bioactive peptides in these products [109].

### 4.6. Supplemented Foods

Foods are not only a source of nutrients; their nutritional content can be further enhanced by the addition of extraneous compounds. In Canada, there exists a separate category of supplemented foods, including energy bars and energy drinks that contain added levels of nutrients (e.g., vitamins, minerals and choline) [110]. Thus, the potential exists for use of bioactive peptides with well-defined physiological roles to be incorporated into commercially available foods and drinks under this regulatory measure. However, this may require robust evidence of biological effect (as demonstrated by studies in human subjects, for example) and consistency before becoming a routine practice. The lack of consistency in natural products like peptides and hydrolysates has long been a limiting factor in better ascertaining their specific roles [111].

### 4.7. Natural Health Products

Not all bioactive peptides are meant to be foods. Many are better suited for use as health products, which can be addressed under the “natural product” category. The growing interest in non-pharmaceutical drugs is a multibillion-dollar industry worldwide, and many bioactive peptides could find successful applications under its umbrella. One of the favorite terms used by both researchers and industry has been “nutraceutical”. A recent search on PubMed for “nutraceutical” had over 3000 hits, with barely 300 for “natural health product” (personal observation, May 2018). However, despite its apparent popularity, the term “nutraceutical” remains poorly defined, with various interpretations and minimal legal/regulatory value [112,113]. From a regulatory perspective, Health Canada uses the term “natural health product” (NHP) and regulates manufacture and sale of such products through the NHP regulations made under the F&D Act [113]. In the United States, the Food and Drug Administration (US FDA) has a similar category of “dietary supplements” [114].

To date, a number of bioactive peptides have been cleared for use under these regulatory systems. The Canadian NHP database already includes “medicinal” ingredients such as casein phosphopeptides, glutamine peptides, and LKPNM (a pentapeptide derived from bonito protein), as well as the hydrolysates of fish protein, lupine, collagen, casein, and shrimp, to name a few [115]. Similarly, the US FDA maintains a list of new dietary ingredients that includes peptides derived from fish, shrimp, sesame, and silk fibroin protein, as well as hydrolysates of egg albumin and milk protein [116]. Given the beneficial roles of bioactive peptides on various physiological systems, this category is likely to be the preferred non-food option for future commercialization.

Interestingly, Schedule 1 of the Canadian NHP regulations specifically mentions (free) amino acids [117]; hence, it could be extrapolated to include synthetic short (2–10 amino acids) peptides in addition. Indeed, non-natural synthesized peptides such as anidulafungin (anti-fungal) and palmitoyl tetrapeptide-7 (skin conditioning agent) are listed in the Canadian NHP database under a separate category [115]. This is a novel option that merits further examination and may become a global model for regulatory approval of synthetic peptides with health benefits.

### 4.8. Pharmaceuticals 

Finally, the most stringent regulatory requirements are reserved for the pharmaceuticals. Unlike food and supplements, pharmaceuticals can be used in invasive routes (e.g., intravenous and subcutaneous) or can be prescribed for specific diseases. However, given the variability in many “natural” products, limited opportunities for patent protection, and the lack of human clinical trials, the pharmaceutical route remains virtually inaccessible to most bioactive peptide formulations [111,118,119,120]. While the potential exists for a handful of synthetic peptides with high potency to break into this field, it would only be realized upon the completion of prohibitively expensive and time-consuming clinical trials, and only for those with robust and unequivocal effects on disease processes. With the legal, financial, and ethical challenges involved, this is unlikely to be a common option in the foreseeable future.

In summary, an understanding of the prevalent and evolving regulatory environment is critical for the success in translating basic discoveries in this field to viable products for improving human health. Given the range of options available, it is advisable to move beyond imprecise and overly broad terminology, and focus on placing novel formulations of bioactive peptides on their appropriate place along the food-drug continuum. Food derived bioactive peptides offer much promise in improving human health, while providing a valuable resource for food producers to prepare value-added products and better utilize the often-wasted by-products. A better understanding of manufacturing, consumer-related, and regulatory aspects would enable broader acceptance and faster utilization of their potential.

## 5. Conclusions

**Figure 1:**
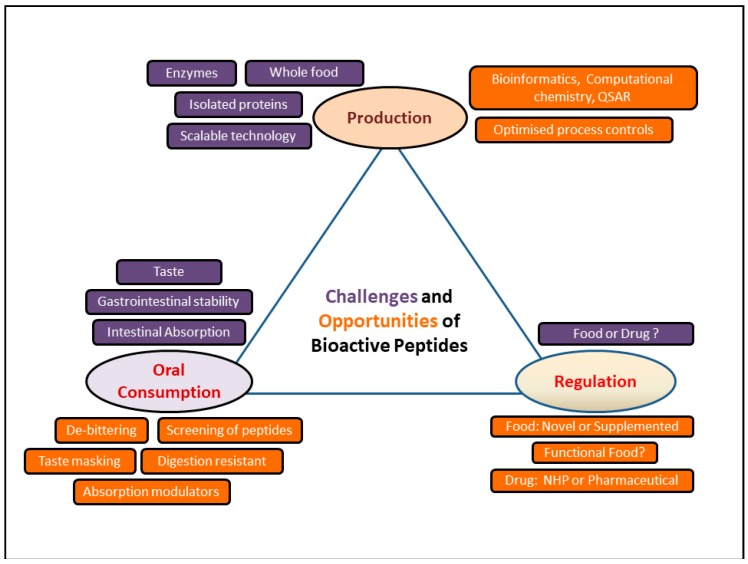
Challenges and potential solutions to utilization of bioactive peptides in human health. The three major aspects attached with the utilizations of food-derived bioactive peptides are (1) production; (2) oral consumption; and (3) regulation. The figure highlights the challenges and future opportunities those are associated with each of the aspects.

Further studies are required for the future use of food-derived bioactive peptides for the prevention and management of chronic diseases. As there are many factors that may influence the production of bioactive peptides, there is still a need to develop a more scalable, affordable, and consistent production technique. The major challenges and future research opportunities associated with the food-derived bioactive peptides are illustrated through a simple line diagram (Figure 1). It is also important to investigate the impact of the co-existing food matrix on absorption of these bioactive peptides. Finally, further research is also required to evaluate the physiological efficacy of these food-derived peptides in human clinical studies. Such scientific knowledge will be helpful for the regulatory bodies to categorize these products, which would facilitate their commercial use to improve human health and wellness.
